# Litter Quality Is a Stronger Driver than Temperature of Early Microbial Decomposition in Oligotrophic Streams: a Microcosm Study

**DOI:** 10.1007/s00248-021-01858-w

**Published:** 2021-09-27

**Authors:** Javier Pérez, Verónica Ferreira, Manuel A. S. Graça, Luz Boyero

**Affiliations:** 1 Stream Ecology Laboratory, Department of Plant Biology and Ecology, Faculty of Science and Technology, University of the Basque Country, UPV/EHU, Bilbao, Spain; 2 MARE–Marine and Environmental Sciences Centre, Department of Life Sciences, University of Coimbra, Calçada Martim de Freitas, 3000-456 Coimbra, Portugal; 3 IKERBASQUE, Basque Foundation for Science, Bilbao, Spain

**Keywords:** Aquatic hyphomycetes, Freshwaters, Leaf traits, Sporulation, Warming

## Abstract

Litter decomposition is an ecological process of key importance for forest headwater stream functioning, with repercussions for the global carbon cycle. The process is directly and indirectly mediated by microbial decomposers, mostly aquatic hyphomycetes, and influenced by environmental and biological factors such as water temperature and litter quality. These two factors are forecasted to change globally within the next few decades, in ways that may have contrasting effects on microbial-induced litter decomposition: while warming is expected to enhance microbial performance, the reduction in litter quality due to increased atmospheric carbon dioxide and community composition alteration may have the opposite outcome. We explored this issue through a microcosm experiment focused on early microbial-mediated litter decomposition under stream oligotrophic conditions, by simultaneously manipulating water temperature (10 °C and 15 °C) and litter quality (12 broadleaf plant species classified into 4 categories based on initial concentrations of nitrogen and tannins). We assessed potential changes in microbial-mediated litter decomposition and the performance of fungal decomposers (i.e., microbial respiration, biomass accrual, and sporulation rate) and species richness. We found stronger effects of litter quality, which enhanced the performance of microbial decomposers and decomposition rates, than temperature, which barely influenced any of the studied variables. Our results suggest that poorer litter quality associated with global change will have a major repercussion on stream ecosystem functioning.

**Supplementary Information:**

The online version contains supplementary material available at 10.1007/s00248-021-01858-w.

## Introduction

Litter decomposition is one of the most important ecological processes globally, as most of the organic matter produced annually escapes herbivory [1, 2] and enters detrital food webs, substantially contributing to the global carbon (C) cycle [3, 4]. Detrital C pathways are particularly important in some ecosystems such as forest headwater streams [5], where primary production is limited by forest shading and nutrient-poor waters [6]. Consequently, these streams are almost entirely fueled by terrestrial plant organic matter inputs, mainly in the form of leaf litter [7], with the transfer of litter C into the aquatic food web being mediated by microbial decomposers, mostly aquatic hyphomycetes [8]. The rate at which microbial decomposition occurs is a key factor influencing the amount of C that is mineralized, incorporated into the local aquatic food web, transported downstream, or sequestered in sediments [9].

Water temperature is a key factor affecting microbial decomposer performance. Warming often enhances microbial metabolism, fungal biomass accrual, conidial production, and microbial-mediated litter decomposition [e.g., 10, 11]. Thus, climate warming is expected to increase the relative contribution of microorganisms to litter decomposition in streams, especially at higher latitudes where microbial performance is temperature-limited [12]. Another factor that can alter microbial-mediated litter decomposition is litter quality, which is expected to be reduced due to changed riparian community composition [13] and also due to increased atmospheric C dioxide (CO_2_) and warming [14, 15]. Additionally, afforestation and other human-related impacts such as the substitution of natural forests by plantations [16], biological invasions [17], or emerging diseases [18] can affect the composition of litter and its decomposition rate, thus altering C flows [19]. In general, these alterations render litter inputs characterized by lower nutrient concentrations, as well as higher concentrations of structural compounds and inhibitory secondary metabolites, which often reduce microbial decomposition [20, 21] compared to softer and more nutritious litter [e.g., 22, 23]. Therefore, it is important to understand how decomposition rates change as a result of such environmental stressors associated to global change.

Given that warming and the increase in atmospheric CO_2_ are occurring simultaneously, it is to be expected that both factors can interact and affect microbial decomposition in non-additive ways. In terrestrial environments, when litter quality and temperature effects have been assessed jointly, substrate quality has been shown to override the effects of temperature on decomposition [e.g., 24]. Also, the decomposition of low-quality litter (i.e., structurally complex C substrates) is more sensitive to a temperature increase than that of high-quality litter [25]. Furthermore, plant chemical and physical defenses remain active after senescence [e.g., 26]. Although there is comparatively less information for decomposition in freshwater than in terrestrial ecosystems, with the latter having greater temperature fluctuations, some studies have suggested that temperature sensitivity of decomposition in freshwaters might also decrease with litter quality [22, 27, 28]. However, such assumption mostly derives from the comparison of a limited number of plant species [e.g., 22] greatly differing in litter quality (e.g., alder vs. oak or alder vs. eucalyptus [23, 29]), or it incorporates other confounding factors such as plant species origin (e.g., native vs. exotic tree plantations [26]). The presence of alder in these studies might have influenced the apparent temperature sensitivity, as previously suggested in a systematic review [28]. Alder is a key component of many riparian communities whose high-quality litter could drive ecosystem functioning and hinder the understanding of underlying mechanisms [30, 31].

Here, we examined the joint effects of water temperature and litter quality on microbial-mediated litter decomposition, and the performance (i.e., biomass accrual, sporulation, and respiration rates) and species richness of fungal decomposers, in a microcosm experiment using litter from 12 plant species and 2 temperatures (10 °C and 15 °C). The species were grouped in 4 litter quality categories based on nitrogen (N) and tannin concentrations, representing different levels of nutrients and defenses. We hypothesized that (i) early microbial-mediated litter decomposition, microbial performance, and species richness would increase with litter quality, due to a release from the deterrent effect of plant defenses and from nutrient limitation in the oligotrophic incubation conditions [e.g., 20]; (ii) microbial decomposer activity would also increase with temperature, given the general metabolism enhancement with warming [32]; and (iii) temperature effects would be lessened for higher-quality litter, given that the decomposition of this litter requires lower activation energy [27, 33].

## Methods

### Leaf Litter Selection and Trait Characterization

We selected 12 plant species covering a range of litter quality, from a dataset obtained in a global study [34] conducted by the GLoBE research network (www.globenetwork.es). The initial dataset contained 146 species from 23 locations distributed worldwide. All species have been characterized based on several key litter traits, and here, we grouped these species into 4 categories that represented different levels of nutrients and defenses, namely the concentrations of N (% dry mass, DM) and tannins (hereafter T; %DM). The 4 categories increased in litter quality according to their N and T concentrations: (1) low N, high T (LN/HT); (2) low N, low T (LN/LT); (3) high N, high T (HN/HT); and (4) high N, low T concentrations (HN/LT) (Fig. S1). Following the same criteria used in a parallel microcosm experiment conducted with detritivores [35], within each category, we selected 3 species to be used in the experiment, with N and T values within the lowest or highest quartiles (Table 1). We avoided species from the same location within a given category, and excluded species from Portugal, where the experiment was conducted, to avoid any potential bias due to ‘home field advantage,’ that is, adaptation of microbial decomposers to local litter [36]. Finally, we excluded alder litter to remove any potential confounding effect of its presence [28, 31].

**Table 1:**
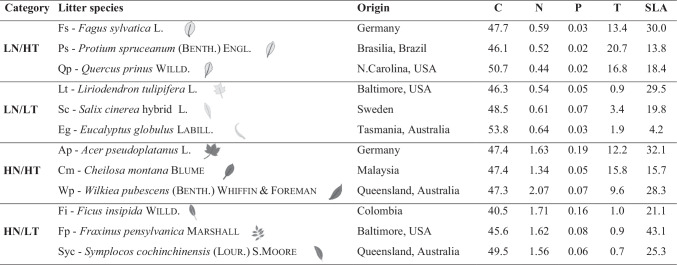
Initial litter traits of the 12 plant species used in the experiment (mean, n = 3), sorted by increasing quality categories in terms of nitrogen (N) and condensed tannin (T) concentrations (LN/ HT: low N, high T; LN/LT: low N, low T; HN/HT: high N, high T; HN/LT: high N, low T). Country of origin (and region in large countries) is indicated, along with litter traits (carbon, C; N; phosphorus, P; and T, all in % DM; and specific leaf area (SLA, in mm^2^ mg^–1^))

For the selected 12 species, we measured concentrations of C and N (%DM) using a CNH auto analyzer (IRMS Thermo Delta V advantage, Thermo Fisher Scientific Inc., Waltham, USA; [37]); T (%DM) with the acid butanol method [38]; phosphorus (P, %DM) with the acid digestion method followed by the molybdate-blue method [37]; and specific leaf area (SLA, mm^2^ mg^–1^) as the ratio of litter disc area to DM (Table 1). For extended details on litter collection and litter trait characterization, see Boyero et al. [34] and Landeira-Dabarca et al. [35]. Additionally, we measured C and N concentration at the beginning of the experiment (i.e., after sterilization). The same sets of discs used to determine mass loss due to leaching during sterilization were used for the analyses (Table S1). Litter discs were ground to fine powder (0.5-mm mesh sieve) and the powder stored in the oven (75 °C) until used. Unfortunately, we were unable to assess P and T concentrations after autoclaving due to the small amount of litter available.

### Aquatic Hyphomycete Inoculum

On March 28, 2014, we collected water and a mixed sample of benthic litter composed of several common deciduous species [mostly willow, *Salix* sp.; chestnut, *Castanea sativa* Mill.; and oak, *Quercus* sp.], at different stages of decomposition, from the Candal stream (Lousã Mountain, central Portugal; 40.08°N, 8.20°W, 620 m a.s.l.). This is a second-order mountain stream that drains an area of 0.98 km^2^ covered by bushes and mixed deciduous forest dominated by chestnut and oak, with very low human activity (0% agriculture and < 2% human settlements) [39]. The stream water is circumneutral, soft, and oligotrophic [39–41]. The litter sample (~ 50 g) was distributed among five 500-mL Erlenmeyer flasks containing 200 mL of filtered stream water, and incubated on an orbital shaker (100 rpm) at 15 °C aided with turbulent aeration provided by air pumps. N and P availability in the medium was increased to promote conidial production (5.5-mg K_2_HPO_4_ and 100-mg KNO_3_ added per liter of stream water, i.e., 1.0 and 13.9 mg L^–1^ of P and N, respectively). This allowed us to obtain a composed fresh conidial suspension (< 1 day old) to be used as inoculum in the microcosms [42].

### Microcosms and Experimental Setup

Just before being used, litter of each selected species was sprayed with distilled water, and litter discs were cut with a cork borer (12-mm ∅). Batches of 10 litter discs (*n* = 6 per species) were dried at 75 °C to constant mass, weighed (± 0.1 mg) to determine initial DM (mg), placed inside glass tubes with 10 mL of distilled water, and autoclaved (20 min at 121 °C). Sterilization removed any possible confounding effects of microflora associated with litter from distinct origins, while at the same time producing effects similar to those of leaching [10]. For each species, 3 extra autoclaved batches of 10 litter discs were reserved as controls to estimate litter mass and N loss due to leaching during sterilization (Table S1) and to calculate a correction factor between initial DM and DM after autoclaving.

Microcosms consisted of sterilized 100-mL Erlenmeyer flasks filled with 40 mL of filtered (Millipore APFF, pore size 0.7 μm, Millipore Corp., Bedford, MA, USA) stream water (Candal stream) and the corresponding litter discs (*n* = 6 microcosm per plant species). Microcosms were randomly allocated to 2 temperature-controlled rooms set at 10 °C (control) and 15 °C (warmed). Control temperature simulated the temperature in Candal stream at the time of microbial inoculum sampling (i.e., early spring, when recalcitrant leaf litter species are still in the benthos and additional litter originate from marcescent tree species or from lateral inputs from the forest soil), while the 5 °C increase in the warmed treatment fell within the upper confidence interval of the A2 scenario within IPCC predictions for the transition to the twenty-second century [43]. Microcosms were agitated on orbital shakers (100 rpm) for 24 h to allow further leaching. The medium was then renewed and each microcosm was inoculated with ca. 3000 conidia of 13 different species (day 0, Table 2). Following standard procedures [42], during the first 6 h, microcosms were agitated for periods of 30 min interspaced with periods of 30 min in which the conidia were allowed to settle; after this, microcosms were agitated continuously until the end of the experiment. The medium was replaced after the first 24 h and thereafter twice a week for the duration of the experiment (1 month), using filtered water (Millipore APFF, pore size 0.7 μm, Millipore Corp., Bedford, MA, USA) collected weekly at Candal stream. All microcosms (*n* = 72, 2 temperatures × 4 litter quality categories × 3 plant species × 3 replicates) were sacrificed at day 31 for conidial and disc sampling. All manipulations of microcosms took place under aseptic conditions to avoid cross-contamination.

**Table 2:**
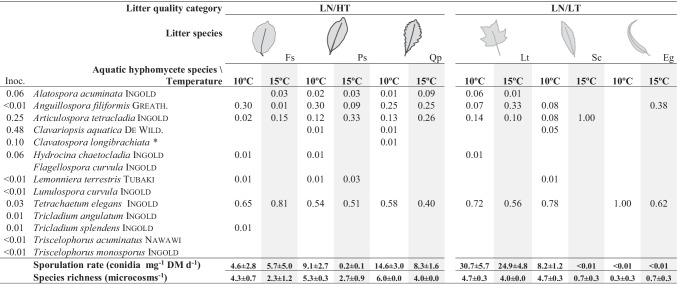

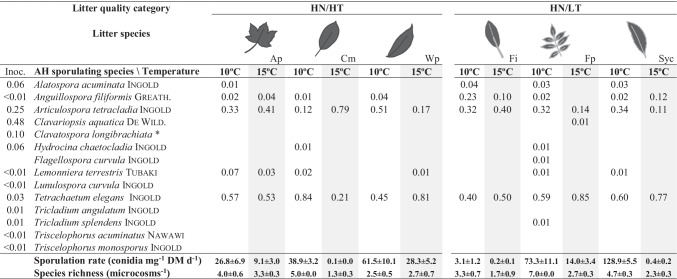
Relative contribution of aquatic hyphomycete species (prop.) to total conidial production associated to different litter species and water temperature (control = 10 °C and warmed = 15 °C, highlighted in gray) (mean, n = 3). Inoc. column shows the proportional contribution of each specie to the inoculum. Litter quality categories, N = nitrogen, T = tannin: low N, high T (LN/HT); low N, low T (LN/LT); high N, high T (HN/HT); and high N, low T concentrations (HN/LT). Total sporulation rate and species richness are given at the bottom of the table (mean ± SEM, *n* = 3)

### Litter Decomposition

From the 10 litter discs in each microcosm, a subset of 5 discs was dried (75 °C to constant mass), and weighed (± 0.1 mg) to determine the DM remaining (mg). The other 5 discs were used to estimate microbial oxygen (O_2_) consumption (i.e., microbial respiration) and fungal biomass (see below). Litter mass loss (%) after 31 days of incubation was calculated as the difference between initial DM (i.e., after sterilization) and final DM remaining (considering all 10 discs) multiplied by 100. The linear decomposition rate was calculated dividing litter mass loss (%) by the number of days (% litter DM d^–1^).

### Microbial Respiration

The second subset of 5 litter discs from each microcosm was used to determine microbial respiration (i.e., O_2_ consumption rate) at day 31, using a closed 6-channel dissolved O_2_ measuring system (Strathkelvin 929 System, North Lanarkshire, Scotland) connected to a computer. The O_2_ electrodes were calibrated against a 0% O_2_ solution (2% sodium sulfite in 0.01-M sodium borate) and a 100% O_2_-saturated microcosm medium, at the target temperature (10 or 15 °C). Litter discs were incubated in 3-mL chambers containing 100% O_2_-saturated microcosm medium with constant stirring (using magnetic stirring bars), and kept at the target temperature by circulation of water originating from a temperature-controlled water bath. After a 1-h trial, litter discs were frozen, freeze-dried, weighed (± 0.1 mg) to determine DM (mg), and used for fungal biomass determination (see below). Respiration rates were determined as the difference in the O_2_ concentration in the control and the sample over a 20-min interval during which O_2_ consumption was linear, and corrected for the chamber volume, time, and disc mass. Results were expressed as mg O_2_ g^–1^ litter DM h^–1^.

### Fungal Biomass

We extracted ergosterol (a surrogate of fungal biomass [44]) from the same subset of 5 litter discs used to assess microbial respiration. Freeze-dried litter discs were placed in tightly closed tubes with 10 mL of KOH/methanol in a water bath (80 °C) for 30 min. The extract was then purified by solid-phase extraction (Waters Sep-Pak® Vac RC tC_18_ cartridges; Waters Corp., Milford, Massachusetts, USA). Ergosterol was quantified by high-performance liquid chromatography (HPLC) by measuring absorbance at 282 nm. The HPLC system (Dionex DX-120, Sunnyvale, CA, USA) was equipped with the LiChroCART 250–4 LiChrospher 100 RP-18 (5 μm) column (Merk, Darmstadt, Germany), and maintained at 30 °C. The mobile phase was 100% methanol, flowing at 1.4 mL min^–1^. Ergosterol was converted into fungal biomass assuming 5.5-μg ergosterol mg^–1^ fungal DM [45] and the results were expressed as milligrams of fungal biomass g^–1^ litter DM [42].

### Aquatic Hyphomycete Conidial Production

At day 31, conidial suspensions were saved into 50-mL Falcon tubes, preserved with 2 mL of formalin (37%) and the volume adjusted to 45 mL with distilled water. When preparing filters for conidial counting, 100 μL of Triton X-100 (0.5%) were added to the suspension, stirred to ensure a uniform distribution of conidia, and an aliquot was filtered (Millipore SMWP, pore size 5 µm, Millipore Corp., Bedford, MA, USA). Filters were stained with trypan blue (0.05%) in lactic acid (60%), and aquatic hyphomycete conidia were identified [46] and counted under a compound microscope at 250 × . Sporulation rates were expressed as number of conidial mg^–1^ litter DM d^–1^ and species richness as the number of species microcosm^–1^.

### Data Analyses

Differences among treatments in microbial-mediated decomposition rate, microbial respiration rate, fungal biomass, and aquatic hyphomycete sporulation rate and species richness were assessed with mixed ANOVA models, with litter quality category and temperature as fixed factors and species nested within litter quality category, acting as random factor, followed by post-hoc comparisons (Tukey tests). The explanatory strength of each factor or interaction in the model was estimated by means of sums of squares type III and presented as a percentage. Data normality and homoscedasticity were assessed with Kolmogorov–Smirnov and Levene’s tests, respectively. Fungal biomass and sporulation rate required a log(x + 1) transformation to meet the ANOVA assumptions.

To help explaining any differences observed in the response variables (excluding species richness, as it was not continuous), we performed Pearson correlation tests not only with initial N and T, but also with the other initial litter traits not used to define litter quality categories (i.e., P and SLA). This allowed us to consider interspecific differences within a litter quality category and the importance of other litter traits. Finally, to compare the effects of warming among these response variables, we examined log-response ratios [log(15 °C/10 °C)], with effects being significant when the 95% confidence interval (CI) did not include the zero value.

The analyses were run on IBM-SPSS Statistics (24.0), with the significance level (α) being set at 0.05 for all the tests.

## Results

### Litter Decomposition

**Fig. 1:**
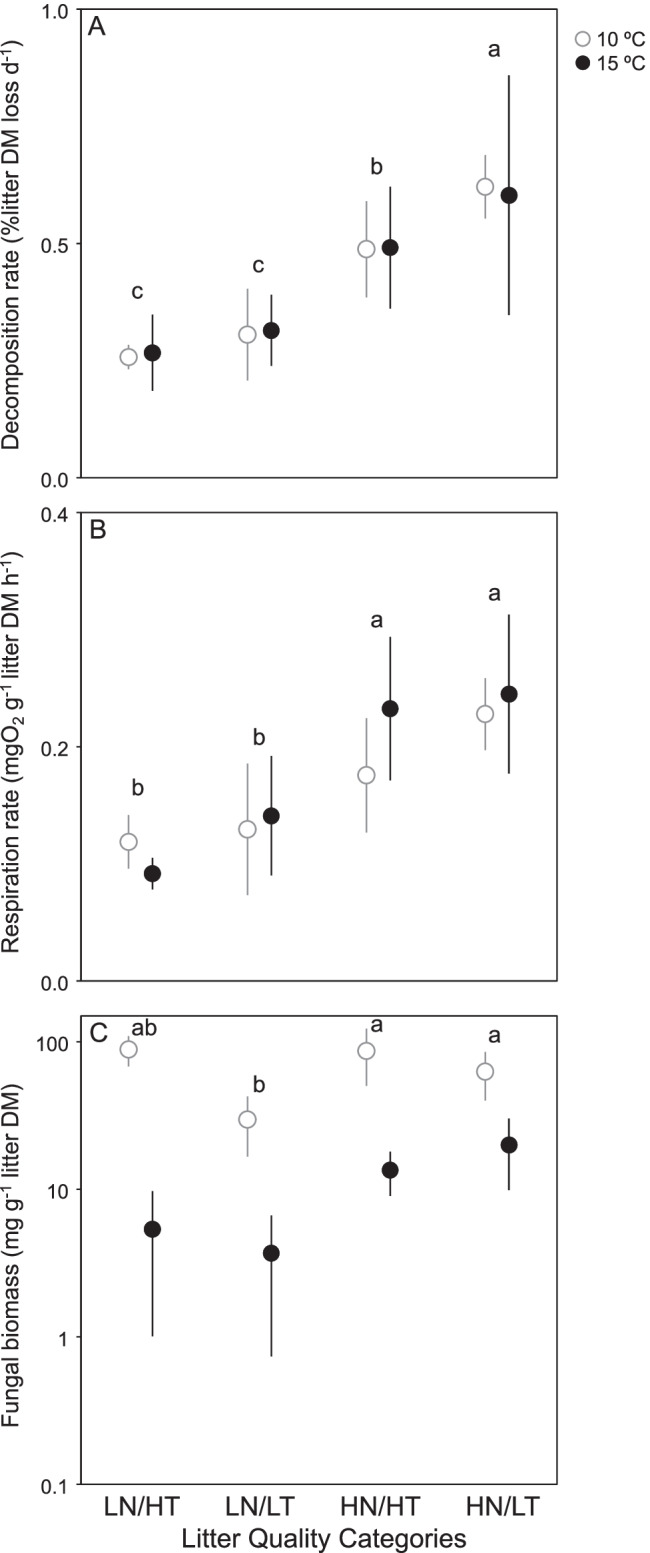
Mean value (*n* = 3 litter species; ± SE) of microbial-mediated litter decomposition (**A**), microbial respiration rate (**B**) and fungal biomass (**C**, log scale) across four litter quality categories varying in nitrogen (N) and tannin (T) concentrations (LN/ HT: low N, high T; LN/LT: low N, low T; HN/HT: high N, high T; HN/LT: high N, low T) and two temperatures (10 °C and 15 °C) after one month of incubation in microcosms. Letters indicate significant differences among litter quality categories (Tukey tests)

After 1 month of incubation, litter mass loss varied widely across the 24 species × temperature combinations, ranging from 5.6% of initial DM for *Eucalyptus globulus* at 10 °C to 29.6% of initial DM for *Ficus insipida* at 15 °C. Litter category had an influence on litter decomposition rates, with mean values increasing with litter quality (Fig. 1A); however, plant species identity within categories was the main factor explaining litter decomposition. Temperature affected decomposition only when interacting with plant species (Table 3).

**Table 3:** Results of mixed ANOVA models, with litter quality category and temperature as fixed factors and litter species nested within litter quality category as random factor (brackets represent the nesting and crosses the interactions). Degrees of freedom (df), *F* test (*F*), and *p* value (*p*) are given, together with the percentage of the variance explained by each component (Var.), estimated by means of type III sums of squares

Variable and source of variation		*df*	*F*	*p*	Var. (%)
**Litter decomposition rate**					
Temperature		1	0.001	0.974	< 0.01
Litter quality category, LQC		3	29.104	< 0.001	32.77
Litter species (LQC)		8	13.324	< 0.001	40.01
Temperature × LQC		3	0.048	0.986	0.05
Temperature × litter species (LQC)		8	3.002	0.009	9.01
**Respiration rate**					
Temperature		1	2.514	0.120	0.84
Litter quality category, LQC		3	28.839	< 0.001	29.05
Litter species (LQC)		8	14.517	< 0.001	39.00
Temperature × LQC		3	1.754	0.170	1.77
Temperature × litter species (LQC)		8	4.310	0.001	11.58
Fungal biomass					
Temperature		1	50.234	< 0.001	33.53
Litter quality category, LQC		3	5.136	0.004	10.23
Litter species (LQC)		8	2.712	0.016	14.48
Temperature × LQC		3	1.067	0.373	2.14
Temperature × litter species (LQC)		8	2.318	0.036	12.38
**Aquatic hyphomycete species richness**					
Temperature		1	89.374	< 0.001	23.89
Litter quality category, LQC		3	11.823	< 0.001	9.48
Litter species (LQC)		8	15.239	< 0.001	32.58
Temperature × LQC		3	5.286	0.003	4.24
Temperature × litter species (LQC)		8	6.209	< 0.001	13.28
**Aquatic hyphomycete sporulation rate**					
Temperature		1	221.914	< 0.001	17.35
Litter quality category, LQC		3	67.565	< 0.001	15.85
Litter species (LQC)		8	43.432	< 0.001	27.17
Temperature × LQC		3	68.849	< 0.001	16.15
Temperature × litter species (LQC)		8	31.200	< 0.001	19.52

### Microbial Respiration and Fungal Biomass

Microbial respiration rates ranged from 0.05 O_2_ g^–1^ litter DM h^–1^ for *E. globulus*, independently of the temperature, to 8 × more for *Acer pseudoplatanus* at 15 °C. Microbial respiration rates significantly increased with litter quality, but were not evidently affected by temperature (Fig. 1B, Table 3). On the contrary, fungal biomass was significantly driven by temperature, being ca. 6 × higher in microcosms incubated at 10 °C (mean across litter treatments: 66.3 mg g^–1^ litter DM) than in those incubated at 15 °C (11.2 mg g^–1^ litter DM); it also varied among litter categories and plant species within categories, but not following a quality gradient (Fig. 1C, Table 3).

### Aquatic Hyphomycetes

**Fig. 2:**
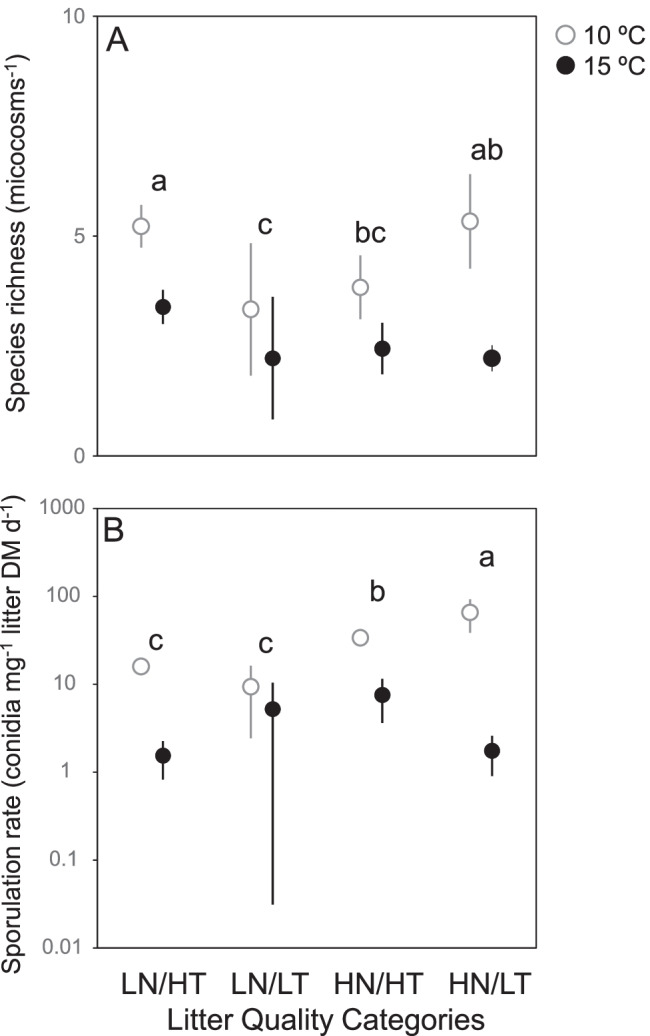
Mean value (*n* = 3 litter species; ± SE) of aquatic hyphomycete species richness (**A**) and sporulation rate (**B**) across four litter quality categories varying in nitrogen (N) and tannin (T) concentrations (LN/ HT: low N, high T; LN/LT: low N, low T; HN/HT: high N, high T; HN/LT: high N, low T) and two temperatures (10 °C and 15 °C) after 1 month of incubation in microcosms. Letters indicate significant differences among litter quality categories (Tukey tests)

A total of 9 sporulating aquatic hyphomycete species were identified, most of them also identified in the inoculum (Table 2). Mean species richness ranged from less than 1 species in *E. globulus*, regardless of temperature, to 7 species in *Fraxinus pennsylvanica* incubated at 10 °C. Species richness was higher at 10 °C than at 15 °C, and there were differences among categories, which did not follow a quality gradient (Fig. 2A). Sporulation rates were higher at 10 °C than at 15 °C and varied among categories, increasing with litter quality (Fig. 2A, Table 3).

Species richness and sporulation rates were mainly driven by plant species within categories, followed by temperature (Table 3). *Tetrachaetum elegans* Ingold and *Articulospora tetracladia* Ingold were dominant or codominant in most of the treatments (Table 2), without any evident pattern explaining the differences among assemblages or in relation to the inoculum.

### Litter Traits Effects

**Fig. 3:**
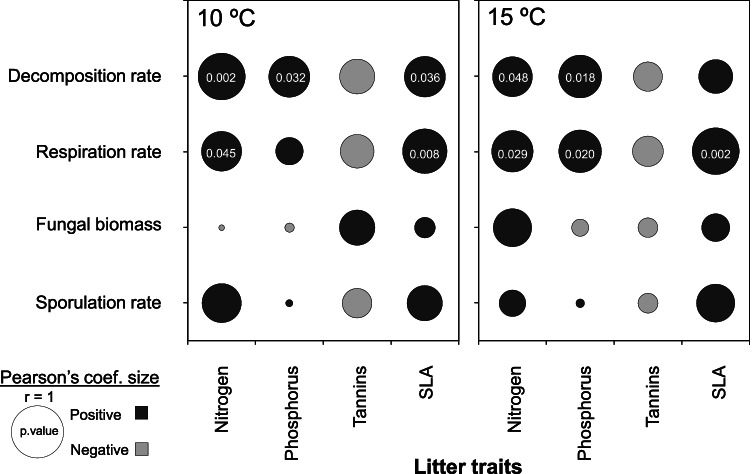
Graphical representation of Pearson’s correlation analysis (*r*) between continuous response variables and litter traits (*n* = 12 species), split by temperature (different plots). Significant correlations are indicated by their *p* values (*p* < 0.05)

The assessed series of correlation analyses helped us to visualize the effects of litter traits on the response variables (Fig. 3). Decomposition and respiration rates were generally positively related with litter N and P concentrations and SLA. Fungal biomass was not explained by any litter trait. Sporulation rates tended to be positively related to litter nutrients (especially N) and SLA and negatively with T, as observed for the other studied rates, regardless of temperature, but these relationships were not significant.

### Effects of Increasing Temperature

**Fig. 4:**
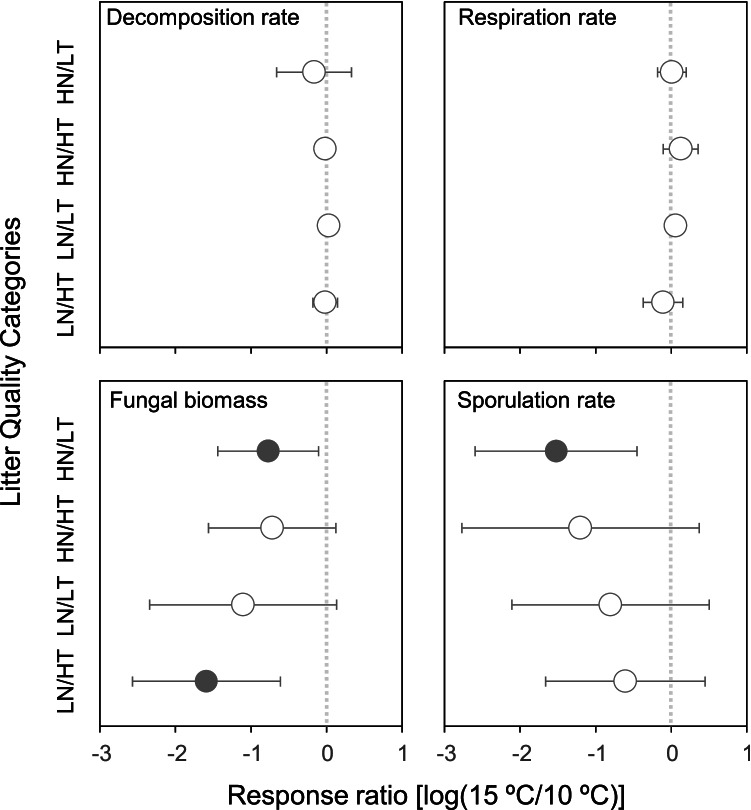
Mean value of the warming response ratios (*n* = 3 litter species; ± 95%CI) within each litter quality category (LN/HT: low N, high T; LN/LT: low N, low T; HN/HT: high N, high T; HN/LT: high N, low T) on continuous response variables (different plots). Close symbols indicate a significant response to temperature increase (i.e., different from zero; *p* < 0.05)

Temperature did not affect litter decomposition and respiration rates, regardless of the litter quality category (Fig. 4). Fungal biomass was generally lower at 15 °C than at 10 °C, but the difference was significant only in the two extremes of the litter quality gradient (HN/LT and LN/HT). Sporulation rates were also generally lower at 15 °C than at 10 °C, but the difference was significant only for HN/LT.

## Discussion

Our microcosm study, conducted with litter from 12 broadleaf species widely differing in key litter traits, indicated that effects of litter quality on stream ecosystem functioning were stronger and more consistent than those of temperature under oligotrophic conditions. Water temperature effects were negligible for early microbial-mediated litter decomposition, and negative for other microbial performance variables (i.e., respiration and sporulation rates, biomass accrual). Litter traits were key drivers of early decomposition phases, with effects that were not apparently related to temperature sensitivity [sensu 28].

### Litter Quality Was the Main Driver of Early Microbial-Mediated Litter Decomposition in Oligotrophic Conditions

Decomposition rate increased with litter quality regardless of water temperature, suggesting that, within the examined temperature range, microbial-mediated litter decomposition was determined by litter intrinsic characteristics. Follstad Shah et al. [28] suggested that some features of stream ecosystems (e.g., water flow and the leaching of secondary compounds and nutrients) could help to explain the weak relationship often found between litter quality and the temperature sensitivity of decomposition, compared to terrestrial ecosystems. These factors could remove or at least mitigate the constraints operating on soils, such as moisture retaining capacity or leachates effects. On the other hand, our experiment took place in early spring, so a 5 °C increase in winter [e.g., 47] might result in an evident warming enhancement of microbial activity. In any case, microbial decomposition seems to be less seasonal in its response to warming compared to detritivore-mediated decomposition [47].

Eucalyptus litter (*E. globulus*) decomposed slowly, despite its low tannin concentration. The slow decomposition of eucalypt litter reported elsewhere has been attributed to its low concentrations of nutrients, high concentrations of secondary compounds including oils and polyphenols, and its waxy cuticle, which could hinder microbial colonization and degradation [16]. We also found low microbial colonization and activity on this species, as shown in other studies [20, 23]. Beech litter (*Fagus sylvatica*), here included in the lowest quality category, also decomposed slowly, as previously reported in field and laboratory studies [48, 49], being specially neglected by detritivores [22]. Among the high-quality categories, *F. insipida* has been reported to decompose at rates similar to alder in temperate sites [49] or even higher in the tropics [50]. As previously stated, forest changes can affect litter composition and decomposition rates, altering C flows [19], and this is not only a projection of climate change effects [13–15], but also natural forests suffering different sources of alternation [16–18]. Furthermore, these changes tend to reduce litter diversity, with repercussions for instream litter decomposition at the global scale [51]. The present study suggests that such replacements will have stronger effects for stream ecosystem functioning than temperature changes, in oligotrophic conditions. For streams with high dissolved nutrient concentration, litter effects on microbial activity and litter decomposition may be mitigated since microbes can satisfy their nutritional needs by uptaking nutrients directly from the water, and therefore will be less dependent on litter nutrients [e.g., 40].

### Temperature Did Not Dictate Early Microbial-Mediated Litter Decomposition in Oligotrophic Conditions

We found that water temperature was not a key driver of decomposition in our experiment. This agrees with some previous studies [e.g., 22, 52, 53], but not others, which have identified temperature as the main determinant of microbial-mediated litter decomposition in field [e.g., 12, 54, 55] and laboratory experiments [e.g., 11, 56], as well as in systematic reviews and syntheses [e.g., 15, 21, 28, 57]. This variety of results suggests that temperature alone is not the main factor determining the activity of microbial decomposers, at least during the initial stages of instream decomposition, and in oligotrophic conditions. It is possible that the stimulatory effect of temperature on litter decomposition was hampered by low dissolved nutrient availability. The interaction between water temperature and dissolved nutrient concentration is complex, but temperature generally has weaker effects on litter decomposition under oligotrophic conditions [e.g., 10]. Also, we only addressed the initial phases of litter decomposition (i.e., 6–30% mass loss), which may have precluded the identification of temperature effects that might need to accumulate over longer periods to become visible. Generally, differences in litter decomposition between environmental conditions increase as time goes [10, 40, 58]. Nevertheless, a portion of the leaf litter entering streams only undergoes initial decomposition in the stream benthos before being incorporated into the sediments during periods of high sediment movement (e.g., spates), or emerged if the stream dries out (e.g., intermitted streams), conditions in which litter decomposition will stagnate, making the assessment of initial litter decomposition most relevant [21]. The temperature sensitivity of microbial decomposers depends on litter traits and decomposability, as shown by a systematic review of stream [28] and terrestrial studies [24]. An increase in litter quality frequently causes a reduction of the apparent temperature sensitivity [e.g., 27, 33, 52]. As previously introduced, in aquatic ecosystems, the presence of alder litter might have biased such assumption [28]. The inclusion of alder in our study could have led to a result different from that found. It is also possible that our single sampling date may have precluded the observation of patterns related with temporal dynamics such as sporulation peaks or successional assemblages [e.g., 8, 23]. This, together with the single inoculation and the short duration of the experiment [59], might explain the lack of effects on conidial assemblages, which might arise in more advanced phases of the decomposition process [23, 60].

### Different Variables Responded Differently to Temperature Increase: from Functional Redundancy to Decreasing Fungal Performance

Higher decomposition rates are often related to increased biological activity such as respiration and fungal biomass accrual [61]. In this study, none of the fungal-related variables increased with warming, which suggests that the examined temperatures remain within their optimal temperature range [sensu 62]. However, this seems to contradict predictions of the metabolic theory [32] and observed latitudinal patterns [e.g., 12], especially when considering the cosmopolitan distribution of fungal decomposers [62, 63]. In our experiment, microbial decomposition and respiration were mainly driven by key litter traits, mostly nutrients, as observed elsewhere [e.g., 27, 64], but fungal-related variables were more variable and apparently affected by warming, as previously observed [56], suggesting higher performance of fungal decomposers at lower temperatures. In a parallel microcosm experiment conducted with detritivores [35], we observed that detritivore-mediated decomposition was scarcely affected by warming, but detritivore performance (i.e., growth rate and efficiency) was boosted by temperature and by the interaction between temperature and litter quality. Taken together, these studies do not support the prediction that microbial contribution to litter decomposition will increase with warming [12, 28, 55], supporting instead an increase in the role of detritivores [47].

## Conclusions

In our microcosm experiment, microbial decomposer activity was influenced more by litter quality rather than by water temperature, which contradicts the general assumption that microbial-mediated litter decomposition is mainly driven by temperature conforming to the metabolic theory of ecology [22, 32, 56]. Our results suggest that litter quality reduction will have stronger effects on the microbial role on stream ecosystem functioning than changes in temperature. Still, warming tended to inhibit fungal decomposers (i.e., reduced sporulation rates and fungal biomass accrual), which could hamper microbial-mediated litter decomposition in the longer term. If our short-term results under laboratory oligotrophic conditions were to be extrapolated to stream ecosystems and extended to a wide geographic scale, then the forecasted changes in riparian vegetation quality concomitant with climate change [13, 14] would reduce the microbial contribution to early litter decomposition, a pattern opposite to its predicted increase due to warming [12]. This suggests that shifts in litter quality need to be taken into account, together with metabolic changes, when predicting global change effects on instream litter decomposition and their repercussions on stream ecosystem functioning [5] and the global C cycle [3].

## Supplementary Information

Below is the link to the electronic supplementary material.

Supplementary file1 (PDF 220 KB)

## Data Availability

Availability of data and material is upon request.
