# Supplementary material for: Litter Quality Is a Stronger Driver than Temperature of Early Microbial Decomposition in Oligotrophic Streams: a Microcosm Study
Source: Microb Ecol. 2021 Sep 27;82(4):897–908. doi: 10.1007/s00248-021-01858-w (PMC8551116; doi:10.1007/s00248-021-01858-w)
Supplement: Supplementary file 1 — Supplementary file1 (PDF 220 KB) [file 248_2021_1858_MOESM1_ESM.pdf]

SUPPLEMENTARY MATERIAL

# Litter quality is a stronger driver than temperature of early microbial decomposition in oligotrophic streams: a microcosm study

Javier Pérez[1,2*], Verónica Ferreira[2], Manuel A. S. Graça[2] and Luz Boyero[1,3]

ORCID

J.P. - 0000-0001-6305-4151

V.F. - 0000-0001-7688-2626

MAS.G.- 0000-0002-6470-8919

L. B. - 0000-0001-7366-9299

[1]Stream Ecology Laboratory, Department of Plant Biology and Ecology, Faculty of Science and Technology, University of the Basque Country, UPV/EHU, Bilbao, Spain.
[2]MARE – Marine and Environmental Sciences Centre, Department of Life Sciences, University of Coimbra, Calçada Martim de Freitas, 3000 – 456 Coimbra, Portugal.
[3]IKERBASQUE, Basque Foundation for Science, Bilbao, Spain.

*Correspondence author: Stream Ecology Laboratory, Department of Plant Biology and Ecology, Faculty of Science and Technology, University of the Basque Country, UPV/EHU, Bilbao, Spain. Phone number: +34 946015939; e-mail address: javier.perezv@ehu.eus

This PDF includes 1 figure and 1 table.

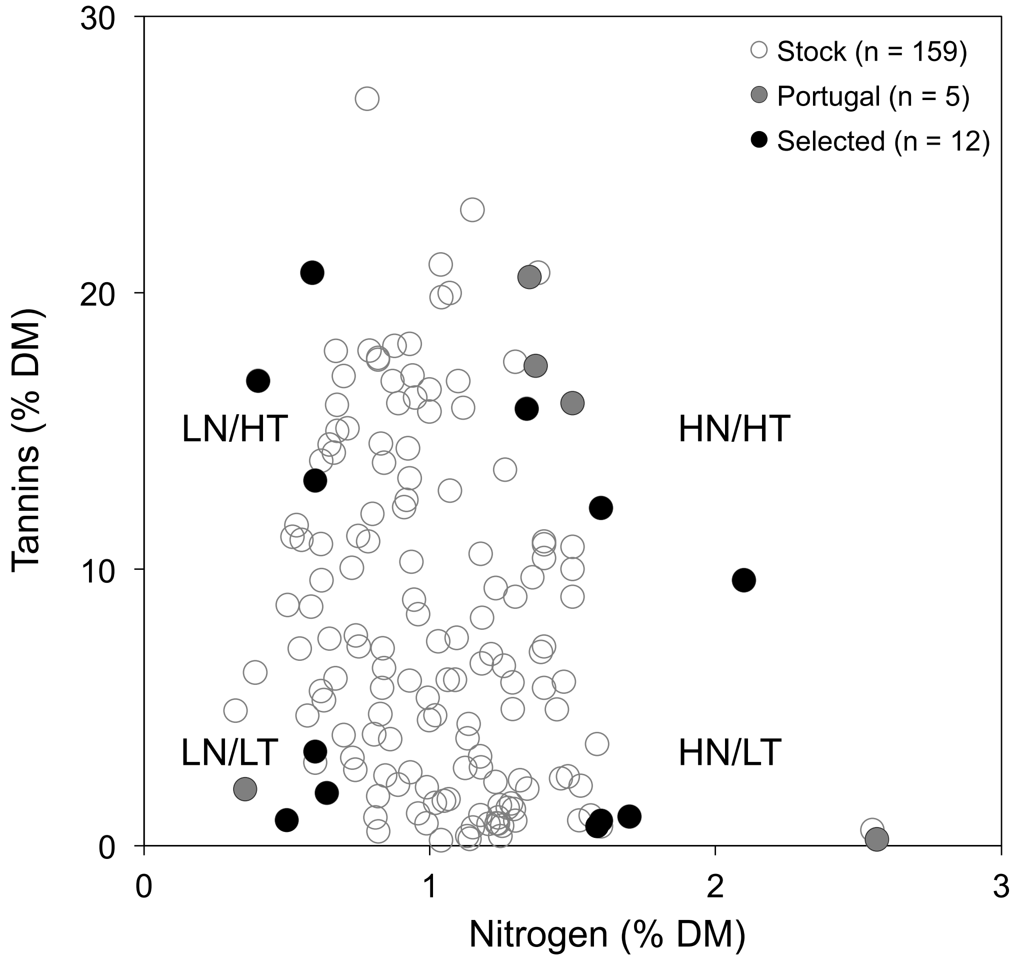


**Figure S1.** Nitrogen (N) and condensed tannins (T) concentration of leaf litter species collected worldwide and the 12 selected litter species in the four litter quality categories: low N, high T (LN/HT); low N, low T (LN/LT); high N, high T (HN/HT); and high N, low T concentrations (HN/LT).

**Table S1.** Estimations of leaching mass loss and the after autoclaving litter N concentration of the 12 litter species (mean ± SEM; n = 3) distributed in the four litter quality categories low N, high T (LN/HT); low N, low T (LN/LT); high N, high T (HN/HT); and high N, low T concentrations (HN/LT).

| **Category** | **Species** | **Leaching (% DM)** | **After leaching N (% DM)** |
|---|---|---|---|
| | *Fagus sylvatica* (Fs) | 8.7 ± 0.5 | 0.54 ± 0.06 |
| **LN/HT** | *Protium sprucianum* (Ps) | 24.3 ± 0.8 | 0.68 ± 0.05 |
| | *Quercus prinus* (Qp) | 10.4 ± 0.3 | 0.64 ± 0.01 |
| | *Liriodendron tulipifera* (Lt) | 39.0 ± 1.3 | 0.69 ± 0.06 |
| **LN/LT** | *Salix cinerea* hybrid. (Sc) | 10.7 ± 0.4 | 0.65 ± 0.05 |
| | *Eucalyptus globulus* (Eg) | 15.2 ± 0.2 | 0.64 ± 0.03 |
| | *Acer pseudoplatanus* (Ap) | 30.2 ± 0.4 | 1.72 ± 0.11 |
| **HN/HT** | *Chelioslea montana* (Cm) | 18.6 ± 0.2 | 1.59 ± 0.07 |
| | *Wilkiea pubescens* (Wp) | 15.2 ± 1.1 | 2.02 ± 0.03 |
| | *Ficus insipida* (Fi) | 15.5 ± 1.6 | 1.91 ± 0.13 |
| **HN/LT** | *Fraxinus pensylvanica* (Fp) | 11.4 ± 0.6 | 1.93 ± 0.16 |
| | *Symplocos cochinchinensis* (Syc) | 26.2 ± 3.4 | 2.65 ± 0.03 |
